# Magnetic Membranes for Cell Growth Under Curved and Reversible Deformations

**DOI:** 10.1002/smsc.202400141

**Published:** 2024-04-30

**Authors:** Valentin Chalut, Damien Le Roy, Thibault Mercier, Marie‐Charlotte Audry, Victor Vieille, Thibaut Devillers, Anne‐Laure Deman, Caterina Tomba

**Affiliations:** ^1^ CNRS, INSA Lyon, Ecole Centrale de Lyon, Universite Claude Bernard Lyon 1, CPE Lyon INL, UMR5270 69621 Villeurbanne France; ^2^ Universite Claude Bernard Lyon 1 CNRS Institut Lumière Matière ILM‐UMR5306 69621 Villeurbanne France; ^3^ Univ. Grenoble Alpes CNRS Grenoble INP (Institute of Engineering Univ. Grenoble Alpes), Institut Néel 38000 Grenoble France

**Keywords:** automatic magnetic actuation, cell viability, magnetic composite membranes, magnetic soft actuators

## Abstract

Magnetic polymer composites are very versatile candidates to fabricate soft robots and actuators thanks to their unique properties such as flexibility and shape memory effect. Thus, the possibility to reproduce natural shapes provides new tools for bioengineering applications. The wide panel of deformations of magnetic polymer composites can be implemented to mimic the movements and curvatures of living tissue. Herein, magnetic polymer membranes are developed to explore cell growth under curved, reversible, and controlled deformations. NdFeB/polydimethylsiloxane composite membranes (86 μm and 46 μm thick) are obtained by soft lithography and magnetized in rolled position under 3 T. Once actuated by a low magnetic field (5–86 mT), the membranes are deformed in wavy shapes with a deformation height of maximum 1.4 and 1.7 mm and a curvature radius of minimum 1.8 and 0.6 mm (86 μm and 46 μm thick membranes, respectively), for a maximum magnetic field of 86 mT. Then, Caco‐2 cell viability is studied on deformed substrates under a static (106 mT) and varying (8–78 mT) magnetic field. No increase in cell death is observed, validating a well‐characterized and promising approach for a new generation of dynamic and curved substrates for cell culture.

## Graphical abstract

Magnetic polymer composites offer unique properties, including flexibility and shape memory effect. In article number 202400141, V. Chalut and co‐workers demonstrated their potential for cell culture on dynamic curved substrates. Soft membranes with wavy deformations were fabricated by a soft lithography process, combining polydimethylsiloxane and NdFeB microparticles, and with no impact on the viability of Caco‐2 epithelial cells.

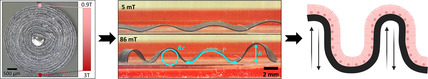

## 1 Introduction

Magnetic composite polymers have enabled numerous advances in the fields of microelectromechanical systems and microfluidics in recent years. Several studies have demonstrated their use in biomedical applications, through cell manipulation^[^ ^1^ ^]^ such as cell sorting,^[^ ^2^, ^3^, ^4^ ^]^ trapping,^[^ ^2^, ^3^, ^5^, ^6^ ^]^ detection,^[^ ^7^ ^]^ or aggregation into magnetic composite tissues.^[^ ^8^ ^]^ The composite approach preserves indeed the properties of polymers while giving them magnetic properties. More specifically, soft magnetic robots have emerged as a technology that takes advantage of remote magnetic actuation of a flexible and deformable material. These robots or actuators are made of magnetic polymers such as Ecoflex^[^ ^9^, ^10^, ^11^ ^]^ or polydimethylsiloxane (PDMS),^[^ ^12^ ^]^ mixed with soft or hard magnetic particles.^[^ ^13^ ^]^ When composed of hard magnetic particles, which can be permanently magnetized, the magnetic profile induced by the application of a magnetic field after polymer cross‐linking gives the composites a shape memory effect. These robots or actuators can memorize and retain specific configurations, and thus reproduce a diverse range of complex programmed movements in a reversible way.^[^ ^10^, ^11^, ^12^, ^13^, ^14^, ^15^, ^16^ ^]^ These properties make them excellent candidates for a wide range of healthcare and bioengineering applications such as microsurgery and diagnostic imaging.^[^ ^17^ ^]^ In particular, as these magnetic robots showed an impressive panel of controlled motions,^[^ ^16^, ^17^ ^]^ they are promising candidates for biomimetic and bio‐inspired technologies for medical applications, such as the delivery of drugs to organs. Magnetic composites have also been recently used to produce actual membranes that apply mechanical stimulations on cells.^[^ ^18^ ^]^

Bio‐inspired approaches have recently been brought into the field of cell culture to study the role of the mechanical properties of the cell environment. In particular, dynamic deformations and mechanical stress may influence cell behavior, whether in terms of survival, proliferation, or differentiation. Works involving mechanical deformation of substrates during cell culture have already been reported in the literature,^[^ ^19^, ^20^, ^21^ ^]^ but only a few of them implement out‐of‐plane deformations. For example, self‐folding substrates, obtained by inducing a preconstraint to one layer of the film^[^ ^22^ ^]^ or bending substrates, deformed by a change in the pressure in the canal below it,^[^ ^23^ ^]^ enabled the recent study of the adaptation of cells to changing curvatures. However, these approaches are based on mechanical deformations that are either not reversible or limited by the stretching of the substrate. Moreover, they generate only one type of curvature (convex or concave) at a time and do not properly reproduce the high complexity and diversity of the human tissue shapes. In the field of soft robots, magnetic polymer membranes (PDMS/carbonyl iron) have been employed to stretch cell monolayers,^[^ ^24^ ^]^ but out‐of‐plane deformation may also be investigated, as wavy membranes of different aspect ratios were reported in the literature. Waves of millimeter wavelength and amplitude up to 0.4 mm were obtained with micrometer‐thick polymer membranes (≈50 μm thick, 1:1 NdFeB/Ecoflex w/w ratio) under a 1 Hz variable magnetic field, ranging from 0 to 3.5 mT.^[^ ^16^ ^]^ Such low magnetic fields can be generated by small magnets, and the resulting deformations were sufficient to move the membranes in a variety of ways, from swimming‐like to walking‐like motions. More recently, Zhang et al. fabricated millimeter‐thick membranes (≈1.5 mm thick, 3:1 NdFeB/PDMS w/w ratio) using a 3D‐printed magnetic composite leading to larger waves of about 15 mm of wavelength and amplitude, under an actuating field of 20–90 mT.^[^ ^14^ ^]^ These results illustrate the possibility of customizing the deformations to suit the purpose, from small deflections inducing displacements to complete folding of the magnetic membrane.^[^ ^16^ ^]^ We, therefore, seek to use the soft‐robots approach to develop a relevant model to mimic the dynamic curvatures of living organisms and to participate in the study of cellular behavior on deformable substrates.

In this article, we report on the fabrication and the characterization of magnetically deformable polymer membranes and on their compatibility with cell growth, with the further aim to study epithelial tissue sensing to bending and to controlled changes of curvature over time. The membranes were made of PDMS loaded with magnetically hard NdFeB particles. By controlling their remanent magnetization profile and the actuating field, it was possible to achieve wavy shapes with variable aspect ratios and deformation amplitudes in the millimeter range. Lastly, to verify that the dynamic substrate is compatible with cell culture and that the magnetic field imposed to deform the membrane does not affect cell viability, we studied the growth and survival of epithelial cells under magnetically induced bending.

## 2 Results and Discussion

### 2.1 Fabrication Process of Hard Magnetic Membranes

**Figure 1:**
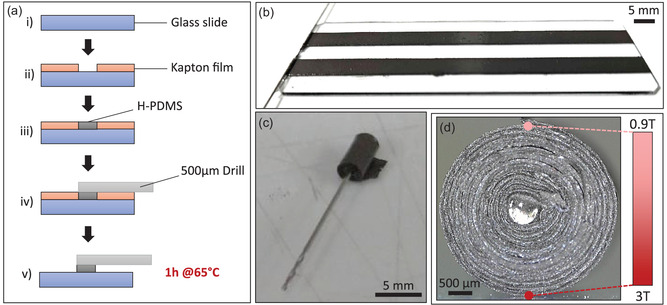
Fabrication process of H‐PDMS magnetic polymer membranes. a) Schematic showing the preparation of H‐PDMS membranes on a silanized glass slide (i), on which two Kapton films (110 μm or 60 μm), sticky on one side, were placed (ii) and a mixture of PDMS and NdFeB (1:4 w/w ratio, i.e., 80 wt% of NdFeB) was spread with a blade between the Kapton films (iii); a 500 μm diameter drill was placed in contact with the mixture at one extremity of the membrane (iv), the Kapton films were peeled off and the sample was cured to 65 °C in an oven for 1 h (v). b) Photograph of the composite after removing the 110 μm thick Kapton films and before the curing step. Two membranes were produced on a single glass slide. c) Photograph of a manually rolled membrane around the metal drill to control the central radius of the rolls. d) Photograph of the side view of a rolled membrane. The rolled membranes were placed on the magnetizing device and exposed to a magnetic field for about 10 μs. The red scale stands for the absolute value of the field that is of 3 T in contact with the magnetizing device and about 0.9 T at the top of the roll.

We employed composite membranes made of magnetically hard PDMS (H‐PDMS), which is a good candidate for combining mechanical softness with magnetically hard properties. To this end, we mixed PDMS with NdFeB powder, leading to a homogeneous distribution of magnetic microparticles (1:4 w/w ratio, i.e., 80 wt% of NdFeB)^[^ ^9^ ^]^ and preserving the property of PDMS to be structured by soft lithography. Following the fabrication process reported in **Figure** **1**a, we used a glass slide structured by Kapton films (Figure 1a‐i‐ii), between which we spread the magnetic material using a blade (Figure 1a‐iii) and cured it (Figure 1a‐v) to obtain a composite membrane (Figure 1a‐iii,b). We employed Kapton films with a thickness of about 60 and 110 μm to develop two categories of membranes with two different thicknesses and therefore different deformation scales. These provided substrates respectively referred to, from now on, as “thin” and “thick” membranes. Then, to give a spatially varying magnetic profile to the membranes, they were rolled (Figure 1c) and magnetized orthogonally to the axes of the roll (Figure 1d), taking inspiration from the magnetization processes used for soft robots.^[^ ^16^ ^]^ The central radius of the roll, which influences the characteristic parameters of the deformation, was set by placing a steel drill with a radius of 250 μm at one extremity of the membrane during the curing step (Figure 1a‐iv‐v). To magnetize the membranes, we employed a pulsed magnetic field generator. This device provides an easy and fast way to magnetize the membranes, and is more adapted for a bench‐compatible fabrication method than superconducting coils which are more constraining tools in terms of operation and implementation. The coil has an inner diameter of approximately 3 mm, which is of the same order of magnitude as the 5 mm‐wide rolled membranes. Thus, the generated field (3 T on its center) used to magnetize the membranes is considered as horizontally homogeneous as a first approximation. However, the generated magnetic field is not homogeneous along the vertical direction and, for a produced field of about 3 T at the magnetizing device surface, its intensity decreases down to about 0.9 T at a distance of 3 mm (data not shown), which corresponds to the diameter of the rolled thick membranes (Figure 1d).

### 2.2 Characterization of the Magnetic Membranes

**Figure 2:**
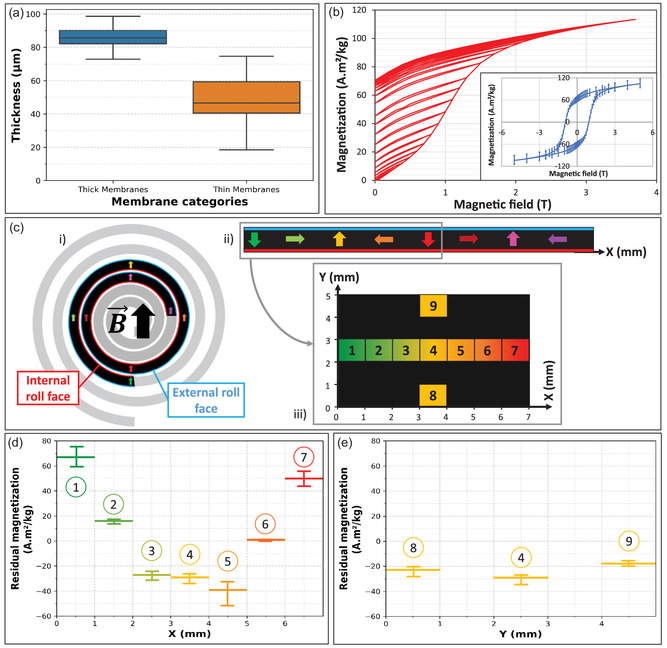
Characterization of the magnetic properties of the membranes. a) Box plots showing the thickness of thick (blue) and thin (orange) membranes. The top and bottom of a box indicate the 75^th^ and 25^th^ quartiles, respectively; whiskers indicate the min and the max values; the middle line is the median. *N* = 3, with 2 maps per membrane by an optical profilometer. Each point of measurement was taken in a set of measurements and datapoints that deviated from the median value of 1.5 × (q3–q1) were considered as aberrant values. b) Magnetic remanence properties of the composite material. First magnetization curve from 0.025 T up to 5 T with regular zero‐field returns. The inset shows the full magnetization curve. Error bars represent the sample weighing error. c) Schematic of the magnetization profile represented by colored arrows at different positions in a rolled membrane (i), which is vertically magnetized, as indicated by the black arrow of the magnetizing field B, giving a spatially varying magnetization profile along the X‐axis shown in the side view (ii); the top view of the membrane (iii) shows the pieces of a magnetized membrane cut along its X and Y axes; the color code stands for their position along the X‐axis. d,e) Residual magnetization along the (d) X and Y (e) axes of a rolled magnetized membrane. The error bars represent the sample weighing error.

After the design and fabrication of the membranes, we characterized their mechanical and magnetic properties. The thickness of the thick and thin membranes (**Figure** **2**a) was measured by optical profilometry. The median thicknesses are 85.6 μm for thick membranes and 46.5 μm for thin membranes, i.e., below the Kapton nominal thicknesses (110 μm and 60 μm). This is probably due to the filling step of the mold. Indeed, H‐PDMS, highly charged in magnetic particles (4:1 w/w ratio, i.e., 80 wt% of NdFeB), is more viscous than PDMS. This limits rapid spreading on the slide and causes the polymer film to shrink relative to the mold. In addition, the mixture is slightly heterogeneous due to the micrometric size of the particles (Figure S1, Supporting Information), which makes the blade coating step even more difficult to control in the case of the thinnest Kapton films. This may explain the higher dispersion in the fabrication of thin membranes. The root‐mean‐square roughness, Rq, was also measured (Figure S2, Supporting Information) using atomic force microscopy (AFM) and, as expected, shows lower values on the surface in contact with the glass (Rq ∈ [14 nm;26 nm]) compared to the upper surface (Rq ∈ [27 nm;67 nm]). Additionally, Young's modulus of a H‐PDMS membrane was measured (Figure S3, Supporting Information) and compared to a similar membrane made of PDMS (1:10 w/w ratio). We obtained Young's modulus of about 5 MPa for the magnetic membrane, which was 5 times stiffer than the one made of PDMS (about 1 MPa). This increase in Young's modulus compared to nonloaded PDMS is expected due to the high concentration of particles inside the polymer matrix.

We measured the magnetic properties of the composite with a Superconducting QUantum Interference Device (SQUID) magnetometer. The room‐temperature magnetization curve, shown in the inset of Figure 2b, shows a magnetically hard behavior with a large hysteresis. The maximum remanent magnetization (*M*_r_) obtained after an exposition to a field strength of 5 T reaches 70 A m^2^ kg^−1^, with an uncertainty of 15 A m^2^ kg^−1^, due to weighing imprecisions. This value is larger than the 53 A m^2^ kg^−1^, obtained after an exposition to 3 T, and 20 A m^2^ kg^−1^, obtained after an exposition to 5 T, of other H‐PDMS (75 wt% and 70 wt% ratios, respectively) reported in the literature, in line with the fact that the composite we use is more concentrated (4:1 w/w ratio, i.e., 80 wt% of NdFeB).^[^ ^12^, ^25^ ^]^ As explained previously, the magnetizing coil produces a magnetic field of 3 T at contact that decreases to 0.9 T (calculated value) at a distance of 3 mm, which is the diameter of the rolled membrane. Given the large hysteresis of the magnetization curve, 0.9 T is not enough to reach a full remanent state. Therefore, to precisely determine the *M*_r_ range of the overall membrane, we measured the first magnetization curves, with successive returns to zero field (Figure 2b). The results show that *M*_r_ ranges from 20 A m^2^ kg^−1^ at 0.9 T to 70 A m^2^ kg^−1^ at 3 T, depending on the distance to the magnetizing coil. Additionally, we observed that, after a period of two months the magnetic moment remained constant, showing good stability of the magnetic profile of the membranes, over a period of at least two months and independently of their initial magnetization (Figure S4, Supporting Information).

The rolling up of the membrane and its magnetization orthogonally to the roll (Figure 2c‐i) provides a spatially varying magnetic polarization along the membrane length (X‐axis Figure 2c‐ii), as reported in the literature.^[^ ^16^ ^]^ The aspect of the magnetization profile could be assimilated to the one of magnetic momentum variation through a Néel domain wall, on a larger scale through the membrane length. To characterize the profile of the membranes after magnetization with the magnetizing device, 1 × 1 mm^2^ pieces of H‐PDMS were manually cut along the two orthogonal axes of a membrane and analyzed with the SQUID (Figure 2c‐iii). During the measurements, the pieces were oriented in the same direction to measure the out‐of‐plane component, along the Oz axis, of *M*_r_. We observed that the projection along this axis of the residual magnetization on a membrane roll (Figure 2d) initially decreases and then increases accordingly with the expectations based on the magnetization profile. Focusing now on the absolute values, the magnetization corresponding to the top of the roll (yellow arrow on Figure 2c), i.e., 39 A m^2^ kg^−1^, is, as expected, lower than the 67 A m^2^ kg^−1^ and the 50 A m^2^ kg^−1^ from the bottom of the roll (respectively green and red arrows on Figure 2c). According to the first magnetization curve, the top part of the roll was probably exposed to ≈1.2 T to get a magnetization of about 39 A m^2^ kg^−1^ while the two bottom parts were probably exposed to a magnetic field ranging from 1.5 to about 2.5 T (respectively red and green arrows). Lastly, at the top of the roll, along the width of the membrane (Figure 2e), the magnetization remains at about −20 A m^2^ kg^−1^ with a variation of 5 A m^2^ kg^−1^, showing that the magnetic field produced by the magnetizing coil is reasonably uniform at a fixed distance to the coil, at the scale of the entire width of the membrane.

### 2.3 Deformation of the Magnetic Membranes

#### 2.3.1 Static Actuating Magnetic Field

The unrolled magnetic membrane can be described as a linear succession of sections that hold magnetic moments with different orientations (Figure 2c‐ii). An external magnetic field will induce a torque on each section's magnetic moment, which yields a mechanical torque on the membrane. In addition, the gradient of the magnetic field will generate a directional force on each section of the membrane, which will be attractive (/repulsive) for sections permanently magnetized parallel (/antiparallel) to the applied field. As a consequence, when submitted to the applied magnetic field and gradient, the membrane will adopt a wavy shape.

**Figure 3:**
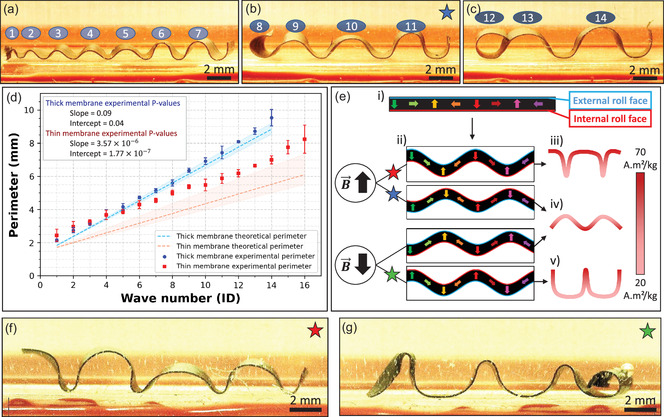
Theoretical and experimental analyses of magnetic membrane shapes under a static actuating magnetic field of about 86 mT. a–c) Pictures on the side view taken with a digital microscope of pseudo‐sinusoidal waves obtained with a thick membrane cut in 3 parts, from the central (a) to the external (c) roll. The cuts of the membranes correspond to the 8^th^ and 12^th^ waves. The blue star refers to the second condition shown in (e). d) Theoretical estimation (dot lines) and experimental values of the perimeter (points) for thick (blue) and thin (red) membranes. The theoretical values of the perimeter were represented using the 250 μm central radius of the metal drill and the median from the thickness measurements. The error band around the dotted line represents the theoretical curve variations taking into consideration the thickness measurement first and last quartiles. Each experimental point stands for the mean value and errors bars denote the standard deviation. *N* = 3, statistical significance threshold *α* = 0.05. e) Descriptive illustration of the shapes achievable through the magnetization process, depending on the relative orientation of the membrane and of the actuating magnetic field B (indicated by the black arrow). Refer to Figure 2c for the color code of the arrows in the membrane sketches. Dark green and red arrows correspond to the regions of higher magnetization than the pink and yellow ones. f–g) Pictures taken with a digital microscope of the side view of nonsinusoidal waves flattened on the top (f) and on the bottom (g) parts. The red and green stars refer, respectively, to the first and fourth conditions shown in (e).

Then, we investigated the wavy deformation of the membranes under an external magnetic field of about 86 mT. Note that this actuating field is greatly inferior to the coercive field and, thus inferior to the field that would reverse the composite's magnetization. As the membranes are rolled around themselves, once unrolled and actuated, they form several waves of pseudo‐sinusoidal shape with increasing length from the central to the external roll (**Figure** **3**a–d). The membranes were cut in three parts, with small (Figure 3a), medium (Figure 3b), or large waves (Figure 3c). We refer to the “height of deformation” to define the distance from the bottom to the top of the waves. From the central to the external roll, the height of deformation increases respectively from 0.4 to 2.8 mm, and 0.4 to 2.4 mm for thin and thick membranes. Since the length of a wave corresponds to the perimeter of a winding, we used the term “perimeter” to describe this parameter. We characterized the perimeter as a function of the wave number (wave n°1 being the first one wound on the rod). To predict its value, the perimeter was theoretically determined (Figure 3d) using the formula (Equation (1)), where *P*_k_ is the perimeter of the wave *k*, *r* is the central radius of the coil (value of 250 μm of curvature radius was taken), and *e* the thickness of the membrane obtained experimentally (Figure 2a).

(1) $$P_{k}=2\pi \left(r+\left(k-1\right)e+\frac{e}{2}\right)$$

Since thin and thick membranes are of the same length, thin membranes allow for more windings, and once unrolled, we obtained 16 and 14 waves, respectively, for thin and thick membranes. As reported in Figure 3d, wave perimeters increase with winding from 2.1 to 8.2 or 9.5 mm, depending on whether they are thin or thick membranes. As expected by the theory, the perimeter for thick membranes is larger than for thin ones, e.g., respectively, 7 and 5.5 mm for the tenth wave. An ordinary least squares analysis was conducted on the experimental measurement points (Figure S5, Supporting Information), confirming a linear regression of the perimeter as a function of the wave number. Moreover, we observed no significant difference between the theoretical and experimental slopes for thick membranes, while a slight discrepancy was observed in terms of intercept (Figure 3d). For the thin membranes, a notable discrepancy emerged, indicating a substantial difference between the theoretical and experimental slopes and intercept. A reason for this observation may be the higher variability obtained for the thickness of thin membranes (Figure 2a) and the difficulty to handle thinner composite membranes at the start of the rolling step and thus to precisely control the inner radius of the winding.

To go further, we observed that the waves formed by the membrane did not have the same shape depending on the orientation of the membrane in the direction of the actuating magnetic field. Indeed, we obtained pseudo‐sinusoidal and nonsinusoidal waves, made of a succession of flattened and narrow regions. In Figure 3e, we have schematized the unrolled membrane with the direction of the rotating induced magnetization, identified its two faces as the “internal” and “external” roll faces (also reported in Figure 2c), and represented the different types of waves obtained. The joint effect of the magnetic force and torque on the magnetically hard particles, embedded in the membrane polymer matrix, must be considered to analyze the generation of the different shapes. At first, by looking at the vertical arrows of the magnetization (Figure 3e‐i,ii), particle magnetic moments oriented in the same direction as the actuating field are attracted toward the maximum magnetic field gradient, i.e., the magnet, while the others are repelled, due to the influence of the magnetic force. Regarding the magnetic torque, the moment of the particles embedded in the polymer matrix tends to align in the direction of the actuating field.^[^ ^26^ ^]^ Consequently, the combination of these effects induces a deformation of the polymer matrix and the undulations of the membrane. As mentioned in Section 2.2, *M*_r_ varies along the membrane length (Figure 2c‐ii), not only in its orientation but also in its intensity (exposed to a magnetic field ranging from 0.9 to 3 T). When the regions of strong magnetization are oriented in the opposite direction to the actuating field, the nonvertical magnetic moments influence the shape of the waves depending on whether they are predominantly oriented in the opposite direction to that of the field (Figure 3e‐iii) or aligned with it (Figure 3e‐v). For these reasons, even if the magnetization profile is pseudo‐sinusoidal,^[^ ^16^ ^]^ the final deformation shows nonsinusoidal waves with a succession of flattened and narrow regions (Figure 3e‐iii,v,f,g). In comparison, when the highly magnetized regions are oriented in the same direction as the actuating field they predominate and the nonvertical magnetic moments have little effect on the shape of the membrane. This resulted in the appearance of pseudo‐sinusoidal deformations for two configurations (Figure 3a–c,e‐iv).

**Figure 4:**
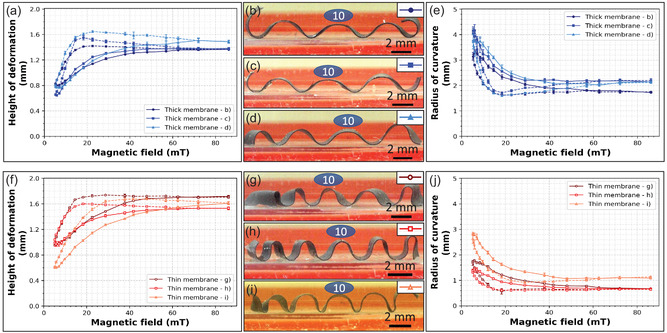
Magnetic membrane deformation under a variable actuating magnetic field. a,e) Mean values of the height of deformation (a) and of the radius of curvature (e) of thick membranes. b–d) Pictures of the side view of thick membranes taken with a digital microscope. The location of the membrane cut produced 3 (b,c) or 4 (d) waves. f,j) Mean values of the height of deformation (f) and of the radius of curvature (j) of thin membranes. g–i) Pictures of the side view of thin membranes taken with a digital microscope. The thin membranes all have 5 waves. The membranes in (g,h) were measured with the internal face of the roll‐up, in the same direction of the actuating magnetic field, whereas in (i), the external face of the roll is up. In the graphs, the error bars denote the standard deviation, the solid lines indicate measurements taken with an increasing magnetic field and the dotted line with a decreasing one; N of consecutive measurements on the same membrane = 3.

Finally, to check the effect of time on the deformation of membranes exposed to a maximal actuating field of 86 mT, the wave height was used as parameter of reference. This value was measured on a membrane (**Figure** **4**b) over a period of 25 h (Figure S6, Supporting Information), and showed a stable height of deformation ranging from 1.35 mm (9^th^ wave) to 1.8 mm (11^th^ wave). We obtained a very stable deformation, with variations of only ≈50 μm, which implies that the deformation quickly reaches its maximum and stays constant, even for several hours.

#### 2.3.2 Variable Actuating Magnetic Field

To produce dynamic substrates that modify their curvature over time, we analyzed the deformation under a variable field B, and we focused on the deformation height (*h*) and the radius of curvature (*R*c) of the top part of the waves (Figure 4). To do this, we brought a permanent magnet close to the membrane, producing a magnetic field of 5 to 86 mT, respectively, at distances of 32 to 6 mm (Figure S7, Supporting Information). The approach was made in 2 mm increments when the distance from the membrane was ≈32 to 12 mm (5–38 mT), and in 1 mm increments when the distance from the membrane was ≈12 to 6 mm (38–86 mT), to improve the precision as the magnetic field quickly increases. To characterize the change in the shape of the membrane, the measurements were carried out in quasi‐static mode. The magnets were moved toward (or away from) the membrane, stopping with a pause time of Δ*t* of 1 s at each position to take a photo of the deformed membrane. We characterized the deformation of the central part of the membrane (Figure 3b). In this portion, the wave shape is more reproducible compared to the extremity regions of the membrane and the magnetic effect of the steel drill is negligible. In the central section, we mainly focused on the central wave, corresponding to the 10^th^ wave. We investigated the deformation of three thick membranes (Figure 4a–e) and three thin membranes (Figure 4f–j).

Figure 4a,e reports the height of deformation and curvature radius of the thick membranes as a function of the applied magnetic field. Interestingly, by slightly shifting the cut of the membrane, just before the 8^th^ and the 12^th^ waves (while the others were cut in the middle of the 8^th^ and the 12^th^ wave), we got 4 waves (Figure 4d) instead of the usual 3 central waves. We thus focused on the central right wave, which corresponds to the 10^th^ wave, as for the waves analyzed in the other two membranes. The curves illustrate a notable augmentation in *h* and diminution in Rc with the increase in the actuating field intensity, reaching a plateau after 60 mT. This deformation is initially maintained when the external magnetic field is decreased, leading to a hysteresis behavior. Under a maximum magnetic field, the three membranes produce comparable deformations, with a maximum height of deformation *h*_max_ ranging between 1.4 and 1.5 mm and a minimum curvature radius Rc_min_ ranging between 1.8 and 2.2 mm. With the thin membranes (Figure 4g–i), we obtained a *h*_max_ ranging between 1.5 and 1.7 mm (Figure 4f), which is similar to the 1.6 mm obtained for the thick membranes. For the membranes shown in Figure 4g,h, the Rc_min_ is of about 0.7 mm. Compared to thick membranes, the curvature radius of thin membranes is about three times smaller.

From a theoretical point of view, the torque and force magnitudes acting on an element of membrane of a given length are proportional to the magnetic moment it holds, and that varies proportionally to its thickness (assuming a uniform magnetization over its thickness).^[^ ^9^ ^]^ Nonetheless, they experience more deformation, due to their reduced resistance to external forces, with a quadratic moment being proportional to the cube of the thickness as *I*_*x*_ = *We* ^3^/12 (with *W* the width and *e* the thickness of a membrane). Therefore, the combination of these phenomena, considered in the torque balance equation,^[^ ^9^, ^10^ ^]^ can explain the larger deformations of thinner membranes. We also measured the differences in the radius of curvature between membranes with pseudo‐sinusoidal and nonsinusoidal deformations, as described in Figure 3f and 4i. The membrane shown in Figure 4i has a lower deformation height and a larger radius of curvature, particularly at low field strength. This result is not surprising due to the flattened shape of the waves. In the context of cell growth on magnetically actuated substrates, introduced in the following part 2.4, the pseudo‐sinusoidal membranes were used as they induce more symmetrical and homogeneous curvature.

### 2.4 Results for a Cell Culture Application

**Figure 5:**
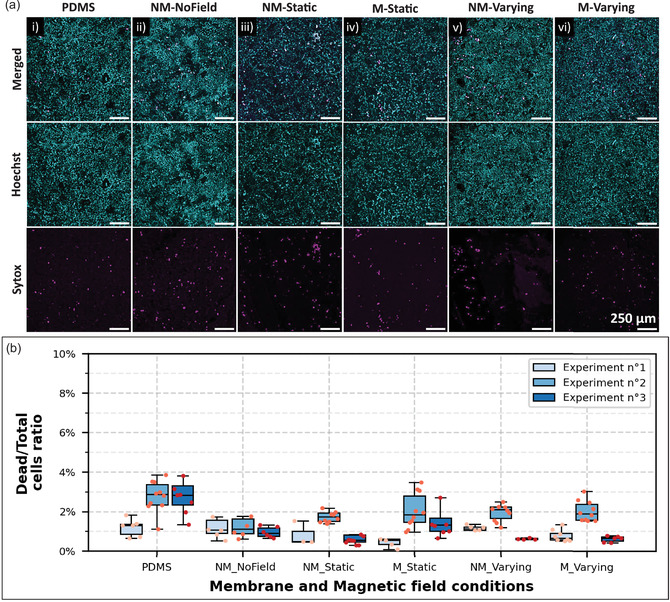
Caco‐2 cells growing on different membranes after 3 days under all magnetic fields conditions and their viability test results. a) Maximum intensity z‐projections at 6 days of culture. Overlay of the Hoechst (nuclei, blue) and Sytox (dead cells, pink) channels of Caco‐2 monolayers: (i) PDMS membrane, (ii) nonmagnetized H‐PDMS (NM‐NoField) without magnetic field, (iii) nonmagnetized and (iv) magnetized H‐PDMS (NM‐Static and M‐Static) under a static field of 106 ± 18 mT and (v) nonmagnetized and (vi) magnetized H‐PDMS (NM‐Varying and M‐Varying) under variable field of 8 to 78 mT. Scale bars = 250 μm. b) Box plots and datapoint showing the ratio of dead to total cell number for each condition for 3 experiments. N of images analyzed varies from 3 to 10 for each condition and experiment. In the box plots, the top and bottom of a box indicate the 75^th^ and 25^th^ quartiles, respectively; whiskers indicate the min and the max values; the middle line is the median. Each point of measurement was taken in a set of measurements and datapoints that deviated from the median value of 1.5 × (q3–q1) were considered as aberrant values.

As we aim to use the magnetic membranes as cell culture substrates, it is necessary to guarantee that such materials and magnetic fields do not perturb cell growth and viability. Several studies investigating the effect of static magnetic fields on cells have been carried out^[^ ^27^, ^28^, ^29^, ^30^, ^31^, ^32^, ^33^ ^]^ and synthesized in specific publications.^[^ ^34^, ^35^ ^]^ For example, static magnetic fields of low intensity (e.g., 6 mT for at least 24 h^[^ ^27^, ^28^, ^29^, ^36^ ^]^) seem to have an influence on cell adhesion and morphology (lipid rafts rearrangement,^[^ ^36^ ^]^ …). More specifically, on a smaller scale, stronger fields (80 mT^[^ ^30^ ^]^ up to 8 T^[^ ^31^ ^]^) were shown to influence molecules presenting diamagnetic anisotropy, such as cytoskeletal structures, actin fibers, and microtubules, inducing cell differentiation, or reorganization. Other qualitative results even showed a potential influence on gene expression, DNA transcription, and translation under similar fields (100 mT^[^ ^32^ ^]^ to 15 T^[^ ^33^ ^]^). However, due to the complexity of cellular systems, the effects of static magnetic fields are unpredictable and vary from one cell type to another, and depend on the intensity and exposure time of the applied field.^[^ ^37^, ^38^ ^]^ We conducted a study on the proliferation and the viability of Caco‐2 immortalized cell line, commonly used as model cells of epithelium. They were grown on different substrates under no field, a static magnetic field (≈106 mT), and a variable field (8–78 mT), generated by a custom magnetic field actuator (Figure S8, Supporting Information). Then, six conditions were investigated (**Table** **1**). Cells were grown on PDMS and nonmagnetized H‐PDMS (NM‐NoField) without magnetic field, to test the influence of the material on cell growth (**Figure** **5**a‐i,ii). Two other conditions, nonmagnetized (NM‐Static) and magnetized H‐PDMS (M‐Static) under a static field (Figure 5a‐iii,iv), were used to analyze the influence of an actuating magnetic field of about 106 mT and of the induced deformation of the substrate (described in part 2.3). Two similar conditions (NM‐Varying and M‐Varying) were employed to investigate the case of a varying actuating field from 8 to 78 mT (Figure 5a‐v,vi). Composed of NdFeB microparticles, also the membranes themselves generate a magnetic field, thereby possibly influencing the surrounding cellular environment. According to the particle's concentration and magnetization curve, this field is of maximum ≈15 mT (estimated on a surface of about 1 × 5 mm^2^), that is an order of magnitude lower than the maximum external field. It should also be noted that the membranes should also give rise to a gradient in the magnetic field at the magnetic particle level. Then, Caco‐2 cells were grown for 3 days on the membranes before being put under the different magnetic field's conditions for 3 more days (Figure 5). The experiment was repeated 3 times and during each of them and for all conditions, cells grew almost reaching surface confluence with an average cell concentration of $2.5\pm 0.7\times 10^{5}$ cells cm^−^^2^.

**Table 1:** Membranes and magnetic field conditions used to test Caco‐2 viability.

	PDMS	Nonmagnetized H‐PDMS	Magnetized H‐PDMS
No field	Control	Effect of H‐PDMS	–
Static magnetic field of ≈106 mT	–	Effect of H‐PDMS and static field	Effect of H‐PDMS, static field, and deformation
Variable magnetic field of 8 to 78 mT with 1 cycle per minute	–	Effect of H‐PDMS and varying fields	Effects of H‐PDMS, varying field, and deformation

Representative images used for the quantification of cell death, for each condition, are shown in Figure 5a. Importantly, no consistent trend was observed across the 3 experiments (Figure 5b), showing a predominant biological variability between samples. Indeed, more significant differences were observed between experiments and samples than between conditions. For all conditions (Figure 5b), the ratio of dead cells to total cells remains relatively low, varying from less than 0.1% to a maximum of 4%, which is a good sign of cell survival. Moreover, the observed cell density after 6 days of culture on the substrates was similar for all conditions, with an average of 2.7 ± 0.7 · 10^5^ cells cm^−^^2^, suggesting a comparable cell proliferation. Thus, these results show that the magnetic field, the material used, or the applied substrate deformation do not decrease cell viability compared to standard PDMS. It is important to note that the quantity of culture medium has to be carefully adjusted to cover the cells also when the membrane is deformed by the magnetic field.

These results confirm that epithelial cells can adhere and grow on our magnetically actuated substrates without impacting their survivability. This means that the approach proposed here to use magnetism to change the shape of the cell substrate over time is compatible with cell culture. Thus, these NdFeB and PDMS composite membranes, deformed under a magnetic field of 8–78 mT, provide a relevant approach for growing cells and studying their behavior on substrates that can be deformed with precise spatial and temporal control.

## 3 Conclusion

In this article, we reported the fabrication of magnetically deformable composite membranes with the aim of developing a new kind of culture substrate. Magnetic polymer membranes, made of NdFeB and PDMS (4:1 w/w ratio, i.e., 80 wt% of NdFeB), were fabricated by blade coating with two different thicknesses (86 μm and 46 μm) and magnetized in a rolled position with a magnetizing device generating 3 T. Once unrolled, such membranes maintain a maximum magnetization of about 70 A m^2^ kg^−1^ and can be remotely deformed in wavy shapes with a low‐strength actuating magnetic field (5–86 mT), produced by a permanent magnet. A maximum deformation height between 1.4 and 1.7 mm and a minimum radius of curvature between 1.8 and 0.6 mm were obtained for thick and thin membranes, respectively, under 86 mT. To demonstrate the value of these deformable substrates for cell culture, epithelial Caco‐2 cells were grown on the membranes for 3 days with no magnetic field and 3 more days under continuous stimulation. No direct effect on cell death of the deformation or the magnetic field, whether static (106 ± 18 mT) or variable (8–78 mT), was observed, demonstrating that these substrates are compatible with cell culture. In conclusion, this approach represents a new generation of substrate to study cell growth under a controlled, reversible, and predictable out‐of‐plane deformation. Beyond this purpose, this strategy for producing physiological culture substrates can also be extended to organ‐on‐chips where the aim is to partially reproduce the biochemical, mechanical, and structural properties of an organ.^[^ ^39^, ^40^ ^]^ This is particularly relevant as most organ‐induced movements and their effects are still poorly understood and mimicked.

## 4 Experimental Section

4.1

4.1.1

##### Fabrication of Magnetic Membranes

To create the membranes, the magnetic composite (H‐PDMS) was fabricated with a mixture of PDMS (10:1 w/w ratio of monomer and curing agent, respectively, Sylgard 184 from Samaro) and powder of NdFeB (0.5‐7 μm size; MQFP‐B from Magnequench International, Inc.), prepared in a mortar in a 1:4 w/w ratio (i.e., 80 wt% of NdFeB). The process to fabricate the substrates is based on soft lithography. Glass microscope slides (25 × 75 × 1 mm^3^; Epredia LR45) were pretreated with 20 μL of Trichloro(1H,1H,2H,2H‐perfluorooctyl) silane overnight under vacuum to make its surface hydrophobic and facilitate membranes detachment. Rectangular shapes (75 × 5 mm^2^) precut on a Kapton film (60 μm; ADEZIF SA 1AZKA822533; or 110 μm thick; Adicaz AHT012L), sticky on one side, were placed onto the silanized glass slides to create a rectangular mold. After this, the magnetic mixture was spread with a blade on the glass/Kapton mold, a steel alloy drill (500 μm diameter) was placed at an extremity of the glass slide in contact with the H‐PDMS membrane, the Kapton film was peeled off the glass slide and then the sample was cured in an oven at 65 °C for 1 h. The curing process finally resulted in a theoretically ≈60 or ≈110 μm high magnetic membrane (respectively named as “thin” and “thick” membranes), and then rolled around the drill. For the thin membranes (60 μm), an extra adhesive tape was added at the opposite extremity of the membrane to prevent it from sticking to itself and to facilitate its rolling‐out. Then, the rolled membrane was placed on a pulsed magnetic field magnetizing device with a coil emitting a pulsed field of 3 T (capacitor charge of 300 V) for ≈15 μs, before being unrolled.

##### Membrane Deformation Analysis Setup

To perform deformations in a controllable way and measure it for different magnetic field values, a setup was 3D printed by an Elegoo Mars 2 Pro printer using an Elegoo standard photopolymer resin and remotely controlled using an ATMEGA328P. It is based on a step motor with an endless screw vertically displacing a platform with a rectangular magnet (NdFeB, 30 × 10 × 5 mm^3^; Supermagnete Q‐30‐10‐05‐N). To observe the membranes in a liquid, similarly to the cell culture conditions, a small container was 3D printed by the same printer and combined with two glass slides to improve the transparency for observations and to reduce the adhesion of the membranes to the substrate. During measurements, the membranes were placed in deionized water 32 mm above the rectangular magnet of the 3D printed setup. Pictures were taken orthogonally to the membrane, with a digital microscope camera (Dino‐Lite Premier Digital Microscope AM4113TL) and DinoCapture 2.0, between each displacement step of the magnet. The magnet was moved from a maximum distance from the membrane of 32 mm (5 mT) in 2 mm steps until it was 12 mm from the membrane, at which point it was moved by 1 mm until the minimum distance of 6 mm (86 mT) was reached. Pause times Δ*t* of 1 s were added after the displacement between each measurement point.

##### Membrane Deformation Setup in the Cell Culture Environment

To perform deformations of the magnetic membranes in a controllable way in the cell culture incubator, the same technique as for the deformation analysis setup was employed. The electronics part was put in a hermetic box to be protected from the high humidity rate of the incubator, while the magnets were left outside of the box. This device was improved to displace four round magnets (NdFeB, 25 × 25 × 5 mm^3^; Supermagnete S‐25‐05‐N) that can be placed just under the four wells of a 12‐well Petri dish. The magnetic field actuation frequency is ≈1 cycle min^−1^ without any pause time or steps and the field ranges from ≈8 to ≈78 mT. Additionally, an ATMEGA328P microcontroller was employed with a sensor to follow the ambient humidity and prevent any electronics dysfunction in the hermetic box. The setup was connected to a cable to be plugged outside of the incubator, as a 9 V battery would be not sufficient to supply the step motor for more than a day. Four round magnets (NdFeB, 30 × 30 × 3 mm^3^; Supermagnete S‐30‐03‐N) were attached on top of the four corners of a 12‐well Petri dish lid. The set was then placed below the 12‐well Petri dish with the samples in the four corner wells, exposing them to a static magnetic field of 106±18 mT.

##### Characterization of the Deformations

A Python code was developed on Spyder to measure the height of deformation and the curvature radius. Briefly, the code enhances the contrast of each image and filters the RGB data, to extract from the image only the black edge of the membrane. Then, the code calculates the difference between the maxima and minima of the waves to determine the height of deformation and uses the chord length formula to obtain the radius of curvature on the top part of the waves. For the height of deformation and curvature radius determination, three membranes were analyzed and for each membrane, three consecutive cycles of actuation were made. The three consecutive measurements were then averaged and the corresponding standard deviation was plotted on each data point. For the perimeter analysis, the same three membranes were measured once and the average values between the membranes for each wave were plotted with the standard deviation. The theoretical curves of the perimeter as a function of the wave number were obtained by using the median thickness and the 250 μm central radius of the metal drill in the formula (1). The error band around the theoretical curve is obtained by considering the variation of thickness between the first and third quartile, respectively, for the lowest and the upmost extremities.

##### Cell Growth and Seeding on Membranes

The membranes, on which were seeded the cells, were exposed to a O_2_ plasma with a PDC 002 CE plasma cleaner for 3 min before being placed in a P100 Petri dish, covered with 100 μL of fibronectin (0.02 μg μL^−1^; Chemicon FC010‐10MG) for 30–40 min at 37 °C, washed with DPBS (10X Diluted PBS Buffer 10X; Biosolve) and placed in a new and clean P100 Petri dish. Human colorectal adenocarcinoma (Caco‐2) cells were grown in DMEM + Glutamax (Gibco Cat# 61965026) supplemented with 10% fetal bovine serum (FBS; Gibco Cat# 10270106), 1% Penicillin‐Streptomycin (PS; Gibco Cat# 15140122), and 1% nonessential amino acids (NEAA; Gibco Cat# 11140035) at 37 °C, 95% humidity, and 5% CO_2_. The medium was changed every 3–4 days and cells were passed once reaching 80–90% confluence. After the trypsinization (TrypLE Express (1X); Gibco 12605‐010), the cells were seeded at 0.4 × 10^6^ cell cm^−2^ by depositing a 100 μL droplet onto the 12 membranes with a surface area of 1 cm^2^. The cells were left for adhesion for about 3–4 h in the cell culture incubator before completing and submerging the substrates in a fresh warm medium. The medium was changed the day after, and the cells were left 2 more days before transferring the membranes to the corner wells of three 12‐well plates. A well plate with 4 substrates on the corners was placed on the support with four round magnets (NdFeB, 30 × 30 × 3 mm^3^; Supermagnete S‐30‐03‐N), another one on the varying field generating prototype and a last one left as control, for an additional 3 days in the incubator. During this period, a change of medium was not necessary. After this period, the medium was removed from the wells and 80 μl of the solution containing (1:2000) Hoechst (Hoechst 33342; Invitrogen H3570) and (1:1000) Sytox (SYTOX; Invitrogen S11380) diluted in culture medium is deposited on each membrane before being left to incubate for 15 min in the incubator. The membranes are then rinsed with PBS and transferred to a P35 Petri dish with fresh medium for imaging. Caco‐2 cells were a kind gift of Aurélien Roux (UniGE, Switzerland).

##### Cell Growth Image Processing

Caco‐2 cells growing on magnetic membranes were observed with either an upright DM6 or inverted DMI8 microscope Leica Thunder Imager 3D Cell culture with a 10x objective (HC PL FLUOTAR 10x/0.32 DRY; Leica 11506521). Z‐stack acquisitions were performed with Z‐steps between 3 and 5 μm. The experiment was carried out 3 times and for each experiment, two membranes per condition were used, except for experiment 1 with only one membrane for the condition of non‐magnetized H‐PDMS with no field. For each membrane, the dead cell/total cell ratio is the average dead cells/total cells obtained for 3–10 pictures taken with the 10x Objective. During observations, the motion of the membranes was restricted by placing a glass coverslip on top of them. Additionally, when using the inverted microscope, the membranes were gently flipped so that cells faced toward the bottom of the petri dish. Images were taken with Z‐Stacks and Z projected with the maximum intensity setting using ImageJ to get rid of the wavy aspect of the membranes and obtain images ready to be analyzed. The pictures were then filtered using a top‐hat filter. The Hoechst channel was thresholded using Bernsen and Contrast auto‐local threshold methods. The Sytox channel was thresholded using Otsu and maximum entropy thresholding methods. The cell nuclei were then detected using the ImageJ analyze particle function. Parameters for the filtering and the thresholding were empirically chosen. The total number of cells was determined using the analyze particle function.

##### Magnetic Characterization Methods

For magnetic characterization, 1 mm^2^ squares of membranes were cut with a scalpel and weighed with an uncertainty of about ±0.03 mg determined experimentally on the weighing scale. About the magnetic properties of the composite material itself, a set of characterizations was made with a SQUID magnetometer (Quantum design) to determine the first magnetization curve, with a regular return to zero‐field to monitor the field‐dependence of *M*_r_, followed by a full magnetization curve, illustrating the behavior of the H‐PDMS under a magnetic field. This characterization consisted in carrying out magnetization cycles. For the profile analysis, once the membranes were fabricated and magnetized using the pulsed field magnetizing device at 3 T, 7 squares were cut along the length and 2 along the width of the membrane between two waves extrema. *M*_r_ of each square was then measured using the SQUID, 20 measurements with two cycles during which the sample was vertically moved by 4 cm down and then 4 cm up from its initial position (twice successively) were performed. Additionally, using the same method, the study of membrane remanence aging was carried out by measuring *M*_r_ of 4 squares (2 from thin and 2 from thick membranes). The same 1 mm^2^ squares were cut into nonmagnetized membranes and then exposed to the 3 T of the pulsed field magnetizing device. The same method was applied to measure the effect of the two directions of the magnetizing fields.

##### Physical Characterization Methods

The membranes were cut into three 25 mm long stripes, except for the perimeter analysis. The thickness was measured using a VEECO optical profilometer. Three thin and thick membranes were bonded by O_2_ plasma to a silicon wafer before being coated with 10–30 nm of Pt–Pd with a Cressington metalliser. They were then analyzed by stitching images obtained with a 10x objective and a 0.5x magnification. Measurements have been carried out two times on three thin and three thick membranes. Each measurement covered a surface of ≈5 × 1 mm^2^ of the membrane. For the roughness measurements, the mean quadratic roughness was determined using AFM (MFP‐3D Asylum Research; Oxford Instruments) equipped with 40 N m^−1^ NCR arrows on regions of 50 × 50 μm^2^. All data were collected using Python through Spyder and analyzed using the scipy.stats library. The NdFeB powder was characterized by scanning electron microscopy (SEM Mira3, Tescan digital microscopy imaging). To carry out Young's modulus analysis, a PDMS and an H‐PDMS membrane (75 × 10 × 1 mm^3^) were subjected to controlled tensile stress to failure with a homemade experimental setup. The corresponding strains were measured and used to calculate Young's modulus of each material.

##### Statistical Analysis

If applicable and as indicated in the legends of the corresponding figures, datapoints that deviated from the median value of 1.5 × (q3–q1) were considered as aberrant values. For the perimeter analysis, an ordinary least square (OLS from Statsmodels 0.14.0) linear regression was used to determine a slope and an intercept from the experimental data points. The OLS was also used to compare the linear regression with the theoretical curve, from which the *p*‐values were extracted. For the cellular viability analysis, a Shapiro normality test (from Scipy 1.10.1) was carried out on the dataset for each category of each experiment, concluding a normal distribution except for the M‐Static of experiment 2. The same test was employed, with the merged experiments datasets leading to the same result. A Kruskal–Wallis test (from Scipy 1.10.1) was carried out to compare the difference between multiple conditions on the merged experiments datasets. All analyses were conducted with Python on Jupyter Notebook (3.11.4, Anaconda, Inc.).

## Conflict of Interest

The authors declare no conflict of interest.

## Supporting information

Supplementary Material

## Data Availability

The data that support the findings of this study are available from the corresponding author upon reasonable request.
