# Supplementary material for: Magnetic Membranes for Cell Growth Under Curved and Reversible Deformations
Source: Small Sci. 2024 Apr 30;4(8):2400141. doi: 10.1002/smsc.202400141 (PMC11934997; doi:10.1002/smsc.202400141)
Supplement: Supplementary file 1 — Supplementary Material [file SMSC-4-2400141-s001.pdf]

Supporting Information

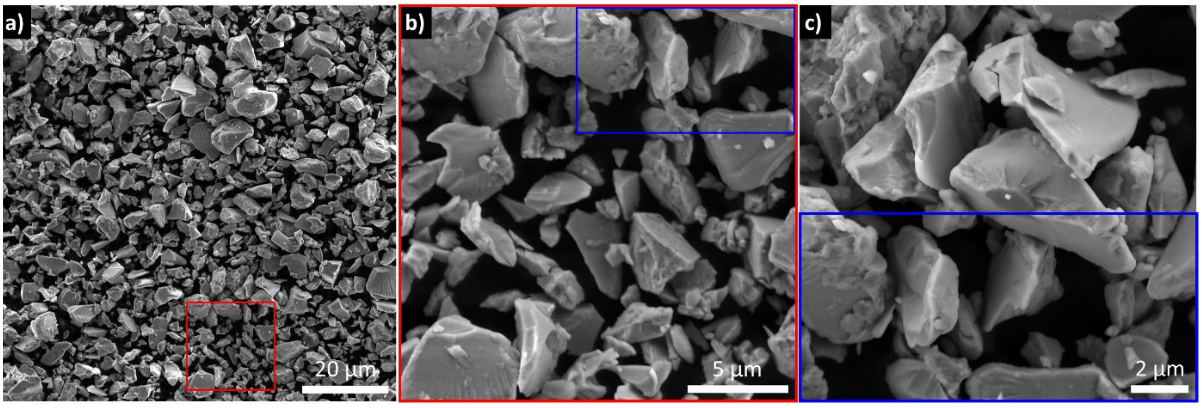

Figure S1. Scanning Electron Microscope (SEM) images of NdFeB microparticles at increasing magnifications from left to right.

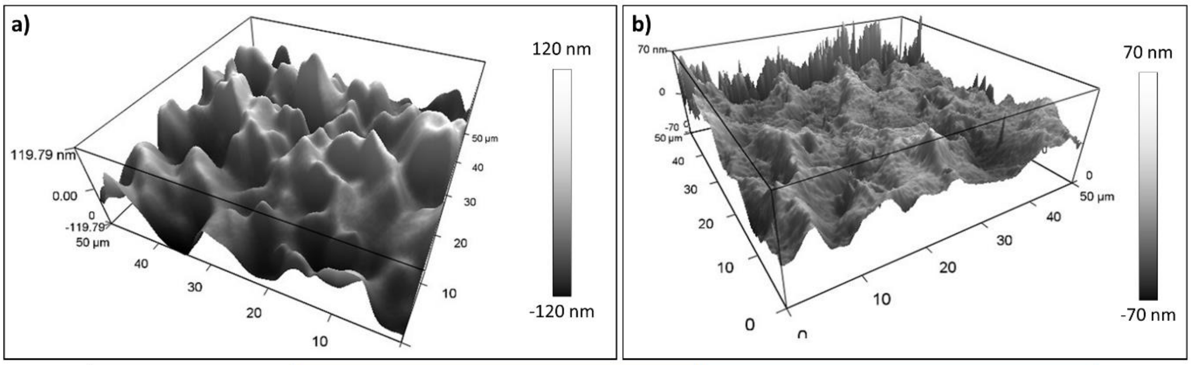

c)	Thick membranes		Thin membranes	
Membrane	Glass side	Air side	Glass side	Air side
N°1	26 nm	50 nm	14 nm	55 nm
N°2	26 nm	40 nm	15 nm	67 nm
N°3	15 nm	27 nm	32 nm	106 nm

Figure S2. Characterization by Atomic Force Microscopy (AFM) in tapping mode. a,b) 3D reconstruction of a 50 x 50 µm^2 surface of a thin membrane on the "upper" (a) and the "bottom" (b) sides, corresponding respectively to the "air" and "glass" sides during the soft lithography process to fabricate the H-PDMS membrane. c) Root mean squares roughness (Rq) values on a 50 x 50 µm^2 surface for the two sides of thick and thin membranes.

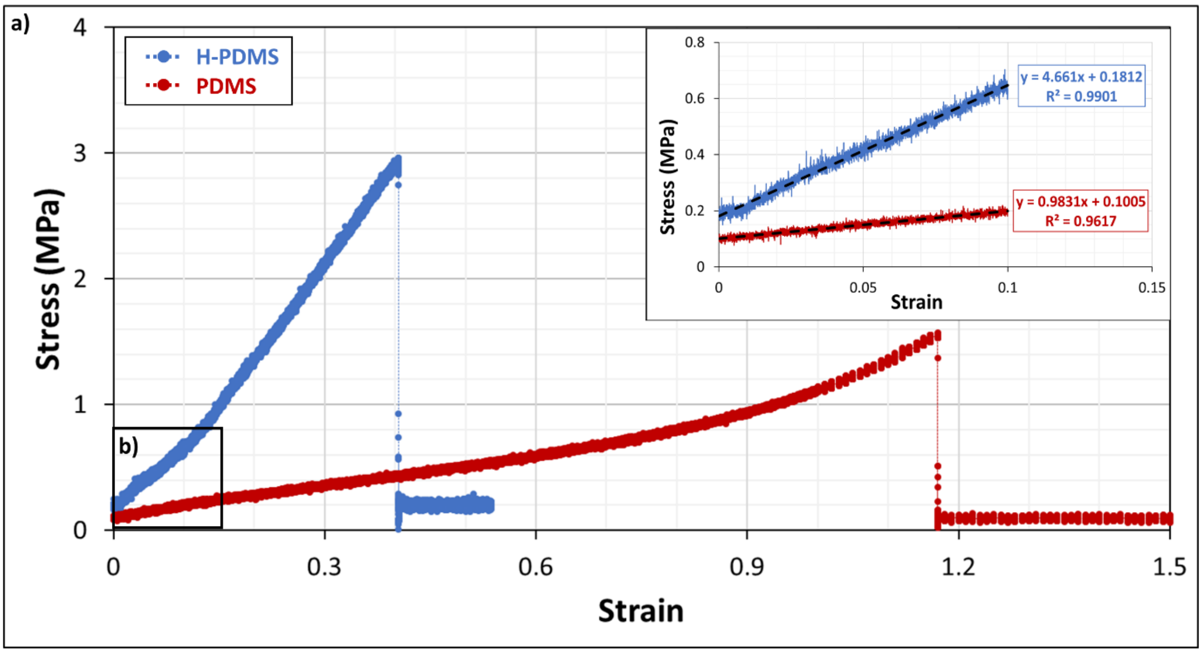

Figure S3. Stress-Strain Curve of PDMS (red) and H-PDMS (blue). Linear elastic region is presented in inset, from which the young modulus of H-PDMS (~ 5MPa) and 1:10 (w/w) PDMS (~ 1 MPa) were extracted.

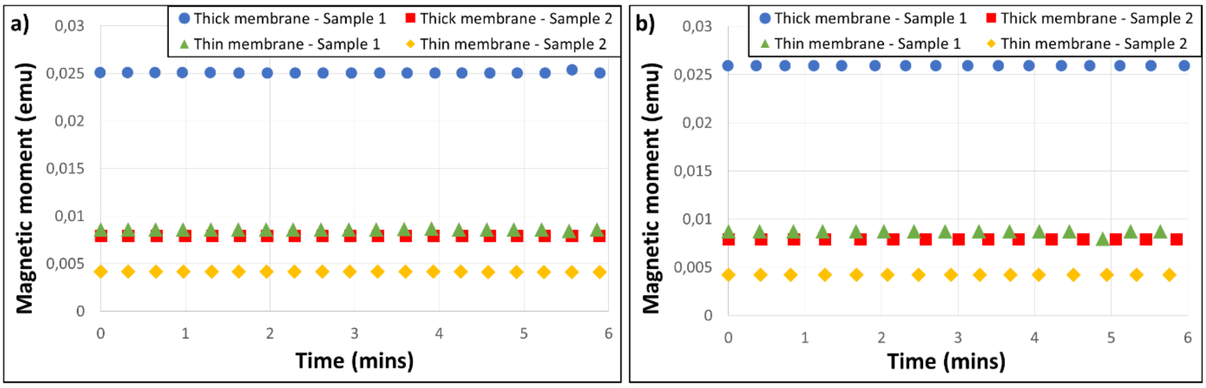

Figure S4. Magnetic moment characterization by SQUID over time. Magnetic moment measurements, over 6 minutes of 1 mm^2 pieces of thick membranes (blue and red symbols) and thin membranes (green and yellow symbols) one (a) and two (b) months after their fabrication and magnetization. Each sample was magnetized with different values of magnetic field.

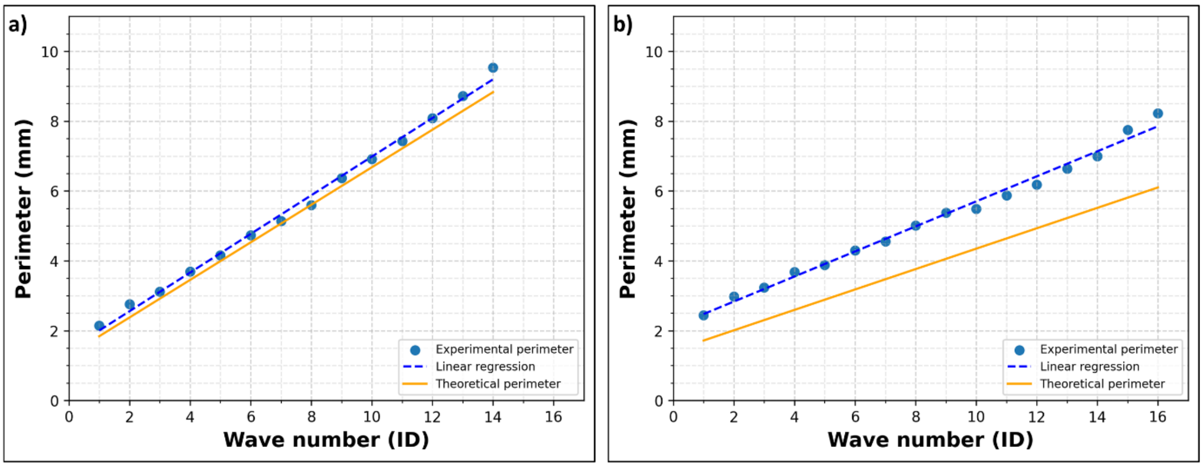

Figure S5. Theoretical estimation (orange) and ordinary least squares (OLS) linear regression (blue dotted line) of the corresponding perimeter experimental datapoints (blue dots) for thick (a) and thin (b) membranes.

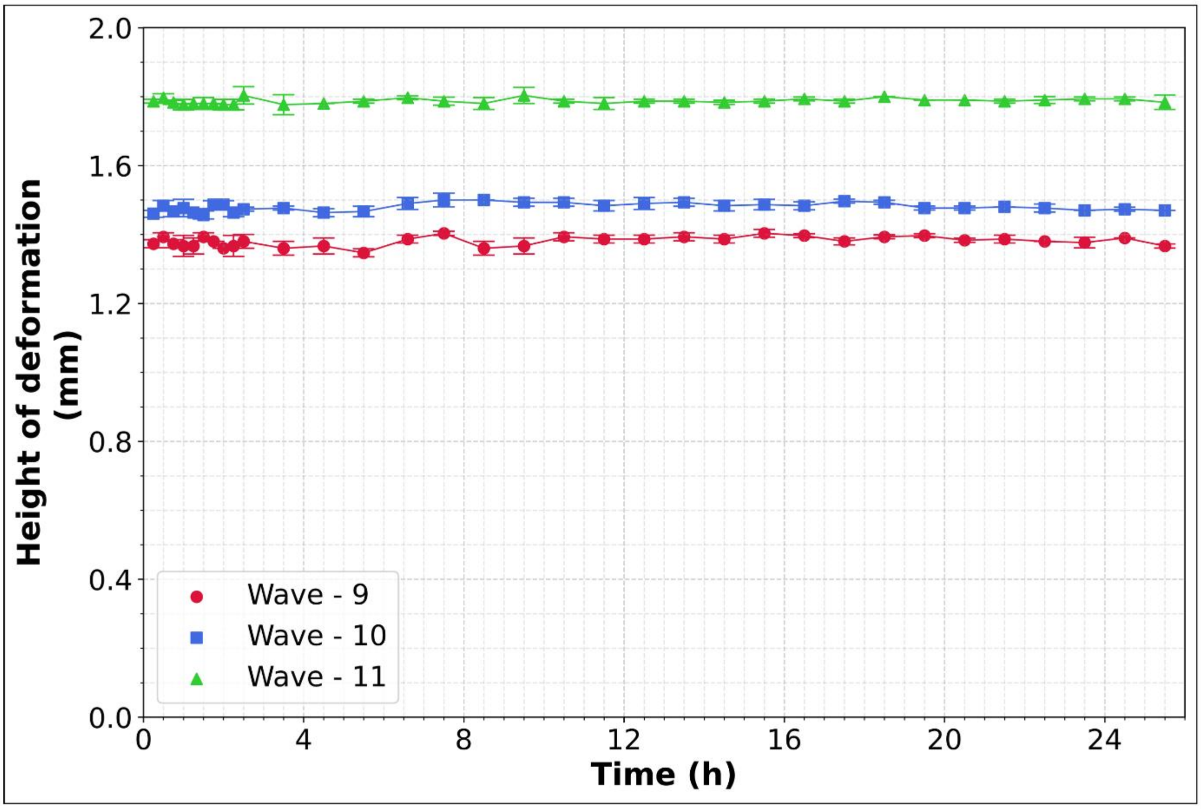

Figure S6. Mean values and standard deviations of the deformation height of a H-PDMS thick membrane (Figure 4b) at a static actuating magnetic field of 86 mT. Measurement acquisitions over time for 26 consecutive hours. N of consecutive measurements on the same membrane = 3.

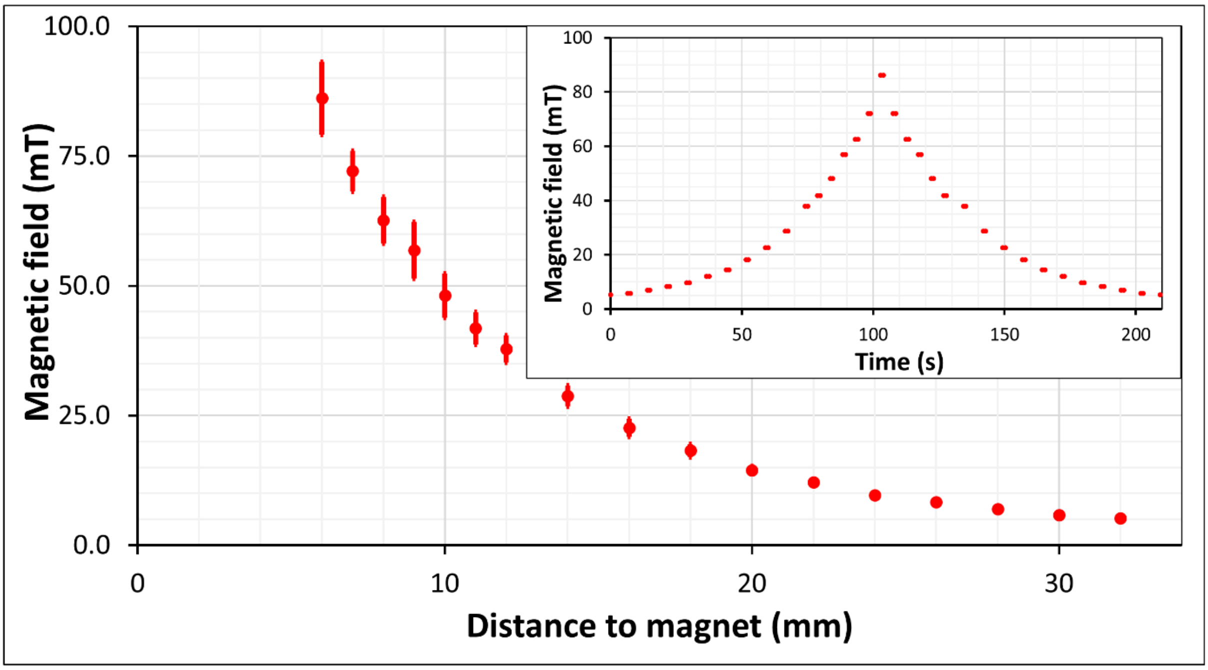

Figure S7. Mean values and standard deviations of the magnetic field as a function of the distance to the rectangular NdFeB magnet. Measurement with a gaussmeter on the center of the membranes normal position. N of consecutive measurements on the same membrane = 6. A cycle of magnetic actuation is presented in inset.

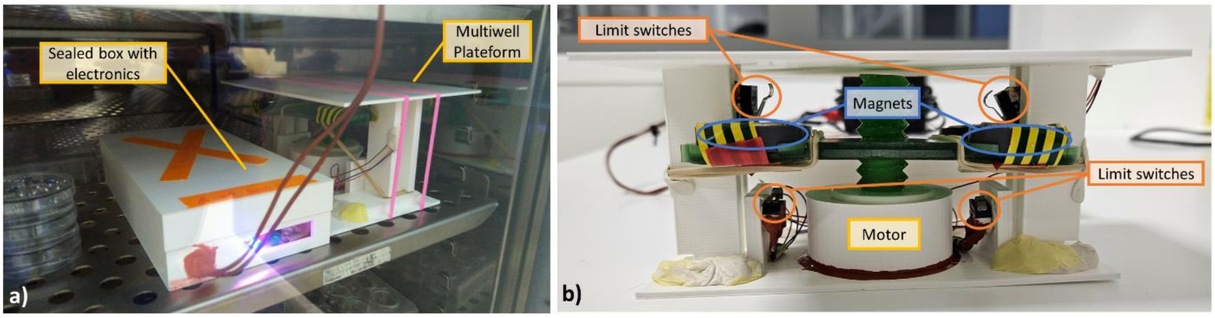

Figure S8. Photograph of the magnetic field actuator for cell culture (a) inside, and (c) outside the incubator.
